# Fatigue Modeling and Numerical Analysis of Re-Filling Probe Hole of Friction Stir Spot Welded Joints in Aluminum Alloys

**DOI:** 10.3390/ma14092171

**Published:** 2021-04-23

**Authors:** Armin Yousefi, Ahmad Serjouei, Reza Hedayati, Mahdi Bodaghi

**Affiliations:** 1 School of Mechanical Engineering, College of Engineering, University of Tehran, P.O. Box, Tehran 11155-4563, Iran; yousefi.armin@ut.ac.ir; 2 Department of Engineering, School of Science and Technology, Nottingham Trent University, Nottingham NG11 8NS, UK; ahmad.serjouei@ntu.ac.uk; 3 Department of Aerospace Structures and Materials (ASM), Faculty of Aerospace Engineering, Delft University of Technology (TU Delft), Kluyverweg 1, 2629 HS Delft, The Netherlands; rezahedayati@gmail.com

**Keywords:** FSSW, finite element model, tensile strength, fatigue life, welding

## Abstract

In the present study, the fatigue behavior and tensile strength of A6061-T4 aluminum alloy, joined by friction stir spot welding (FSSW), are numerically investigated. The 3D finite element model (FEM) is used to analyze the FSSW joint by means of Abaqus software. The tensile strength is determined for FSSW joints with both a probe hole and a refilled probe hole. In order to calculate the fatigue life of FSSW joints, the hysteresis loop is first determined, and then the plastic strain amplitude is calculated. Finally, by using the Coffin-Manson equation, fatigue life is predicted. The results were verified against available experimental data from other literature, and a good agreement was observed between the FEM results and experimental data. The results showed that the joint’s tensile strength without a probe hole (refilled hole) is higher than the joint with a probe hole. Therefore, re-filling the probe hole is an effective method for structures jointed by FSSW subjected to a static load. The fatigue strength of the joint with a re-filled probe hole was nearly the same as the structure with a probe hole at low applied loads. Additionally, at a high applied load, the fatigue strength of joints with a refilled probe hole was slightly lower than the joint with a probe hole.

## 1. Introduction

Lightweight metals such as aluminum alloys are widely used in the automobile and aerospace industries. Assembling such metal structures is a challenging process in the industry. Resistance spot welding (RSW), laser spot welding, and riveting are widely used to assemble aluminum alloy panels. However, the noted conventional methods have several disadvantages, including high structural weight and weak joint strength. Therefore, a new method is required for joining aluminum panels in order to overcome these shortcomings. Friction stir welding (FSW) is a revolutionary joining method developed by TWI (Cambridge, UK) in 1991. This method has various advantages, such as small thermal deformation, high-quality welds with superior mechanical properties, fine and uniform weld microstructures, and high welding efficiency [1].

Friction stir spot welding (FSSW) is a newly generated solid-state joining process [2]. In this process, the specimens are connected due to friction heating on the facing surfaces of a specially designed tool and sheets. The stirring process plays an essential role in the efficiency of the welding process [3]. Nowadays, FSSW has gained considerable interest because of its practical application in the industry, for example, joining metals, such as magnesium, titanium, copper, as well as its ability to join polymers, composites, and dissimilar materials [4,5].

Structures joined using FSSW are subjected to different loading conditions during their life service. Therefore, it is vital to elucidate the tensile and fatigue strengths of these structures. Several studies have [6,7,8,9,10] investigated the static and fatigue performances of FSSW jointed structures. Ogawa et al. [6] investigated the influence of welding process time to improve the FSSW joint efficiency of aluminum alloy. Furthermore, the fracture mechanism was observed thoroughly. The results showed that increasing the welding process time enhances the static and fatigue strengths. Uematsu et al. [7] performed tensile and fatigue tests using lap-shear specimens of dissimilar FSSW between aluminum A6061 and low carbon steel. The results were compared with a similar weld of two A6061 sheets. Results revealed that the tensile strength of a dissimilar FSSW joint is higher than that of a similar one. However, the fatigue strengths of both types of FSSW joints were nearly the same. Lin et al. [8] experimentally investigated the microstructures and failure mechanism of FSSW joints in aluminum 6111-T4 lap-shear specimens joined by two different types of tools (a tool with a flat tool shoulder and another one with a concave tool shoulder) subjected to static load. They observed that the failure is initiated near the stir zone in the weld zone’s middle section. Tozzi et al. [9] investigated FSSW joints between 6061-T4 aluminum alloy sheets. Tensile and shear tests were performed for three different probe lengths. The effect of the probe depth on static strength and fracture mechanism was discussed. Hassanifard et al. [10] studied the fatigue behavior of multi-friction stir spot-welded joints. They examined the effect of FSSW layout on fatigue life. Results indicated that the arrangement of one row of four joints perpendicular to the loading direction is the best as it has highest fatigue strength. Ebrahimpour et al. [11] investigated the TRIP steel sheets jointed by the FSSW process for different rotational speeds experimentally and numerically. FEM was employed to calculate the effects of temperature, strain, and strain rate during the welding process. The results indicated that, by increasing the rotational speed, the temperature, strain, and strain rate increased. The results revealed that increasing the rotational speed (until 1500 rpm) results in increasing the tensile strength, although at higher rotational speed (1800 rpm), the tensile strength decreases.

One of the disadvantages of FSSW joints is the development of holes on the surface at the central part of the weld nugget due to the FSSW process. The presence of the holes causes several problems, such as corrosion vulnerability, in the structure. In fact, water could remain in the holes for a long time, and corrosion occurs in such areas [12]. To address this problem, different methods have been developed to refill the probe hole, such as a double-acting FSSW tool. Re-filling the probe hole results in high weld strength, an adequate load-bearing capacity, and reduction in corrosion vulnerability [12,13,14,15]. Uematsu et al. [12] experimentally investigated the effect of re-filling the probe hole on the tensile strength and fatigue life. The results indicated that the re-filling probe hole results in increasing the static strength. However, re-filling the probe hole has no considerable effect on the fatigue strength. Several recent studies [16,17,18,19,20,21,22,23,24,25,26,27] used different loading conditions and different viable approaches to investigate the tensile strength and fatigue life of joints and metallic structures.

The finite element method (FEM) is a cost-effective method to study FSSW joints. By performing the FEM, the effect of different FSSW process parameters on the tensile strength and fatigue life could be investigated. In this study, the fatigue behavior of Al-Mg-Si aluminum alloy (A6061-T4 aluminum alloy) joints, joined by friction stir spot welding (FSSW), is investigated numerically. Effect of re-filing the probe hole on the tensile strength and fatigue life is examined and compared to the experimental results of Uematsu et al. [12]. It is worth mentioning that to calculate the structure’s fatigue life; the strain-based approach is used. The homogenized variable (strain) is determined by the volume averaging of the strain from all elements in the defined cylindrical-shaped volume (as a representative volume element) around the probe hole. To the best of the authors’ knowledge, no research has been done to investigate the tensile strength and fatigue behavior of FSSW joints based on strain-based approaches by volume averaging variables to achieve more reliable results. In this regard, to calculate the fatigue life, the steady-state hysteresis loops for different applied loads are plotted, the plastic strain is determined, and the fatigue life is calculated. This paper is organized as follows: in Section 2, the materials and geometry of structures joined by the FSSW process are defined. Then, the different steps of the FSSW process are explained. In Section 3, numerical modeling is elucidated, mesh type and mesh size are defined, and boundary conditions are determined. The fatigue prediction procedure is also explained. In Section 4, results and relevant discussions are presented. First, tensile strength results are discussed, and then the fatigue prediction outcome is debated. Finally, a summary is presented, and the conclusions are drawn.

## 2. Materials and Methods

### 2.1. Materials and Design

The sheets used in this study were made of A6061-T4 aluminum alloy (elastic modulus of 68.9 MPa, yield strength of 145 MPa, and tensile strength of 252 MPa [28]) with a thickness of 2 mm. An elastic-plastic material model was employed to model the mechanical behavior of aluminum sheets. The Johnson-Cook model, with constants listed in Table 1, was used to model the plastic behavior of the sheets. The proposed Johnson-Cook model and the constants were assumed to match the material flow behavior of the FSSW joints in the Uematsu et al. study [12].

**Figure 1:**
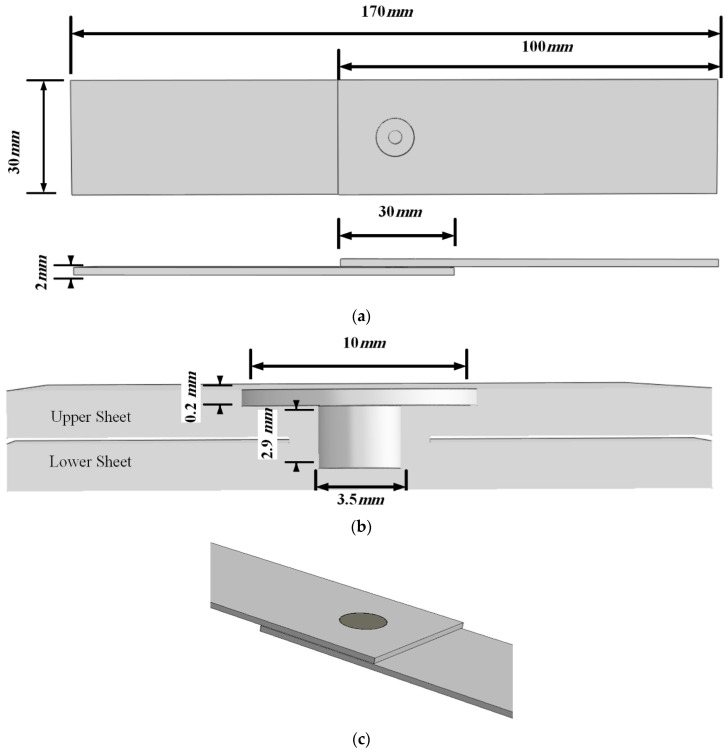
(**a**) Geometry of the specimen made of two sheets overlapping one another, (**b**) geometry of hole, and (**c**) a model with a refilled hole.

**Figure 2:**
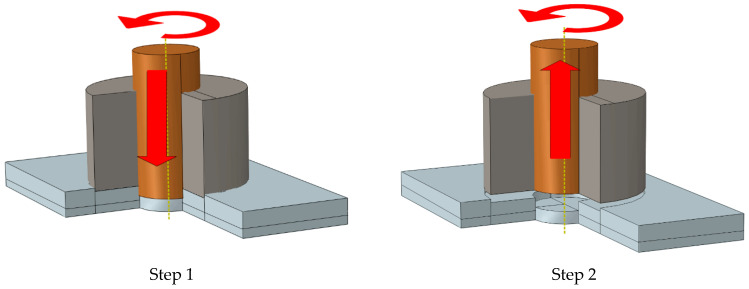

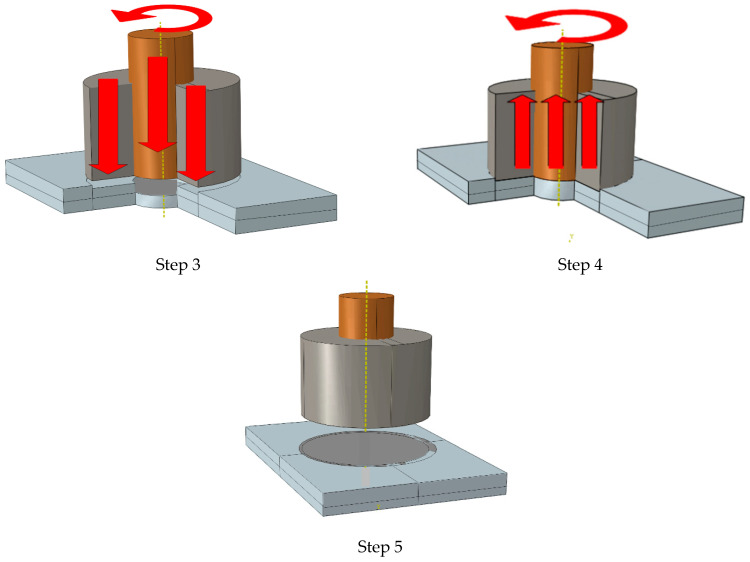
Different steps of FSSW and re-filling process.

Figure 1a shows the geometry of the specimens made by placing two aluminum sheets with dimensions of 30 × 100 mm^2^ on top of each other with a 30 × 30 mm^2^ overlap area. The hole geometry is demonstrated in Figure 1b, which is created by a double-acting tool consisting of a flat outer shoulder and an internal retractable probe, which could refill the probe hole. The geometry of the double-acting tool is the same as the hole created by the tool. Figure 1c shows a model with refilled hole. Figure 2 indicates different stages of the FSSW re-filing process. The tool used in the re-filling process consists of three components: an outer clamping ring, a sleeve, and a central pin. Both the sleeve and pin components rotate at the same rotational speed and direction [30]. In the FSSW process, in the first step, both the central rotating pin and outer rotating clamping ring move down and impose compressive pressure on the contact surface (Step 1 in Figure 2). In the second step, the central pin moves up (Step 2 in Figure 2). Then, both the central pin and the outer clamping ring move down again to fill the probe hole with heated and softened material (plasticized material) due to the friction between the metal surface and the unconsumable pin (Step 3 in Figure 2). After re-filling the probe hole, the rotating FSSW tool moves up (Steps 4–5 in Figure 2) [31].

### 2.2. Numerical Modeling

**Figure 3:**
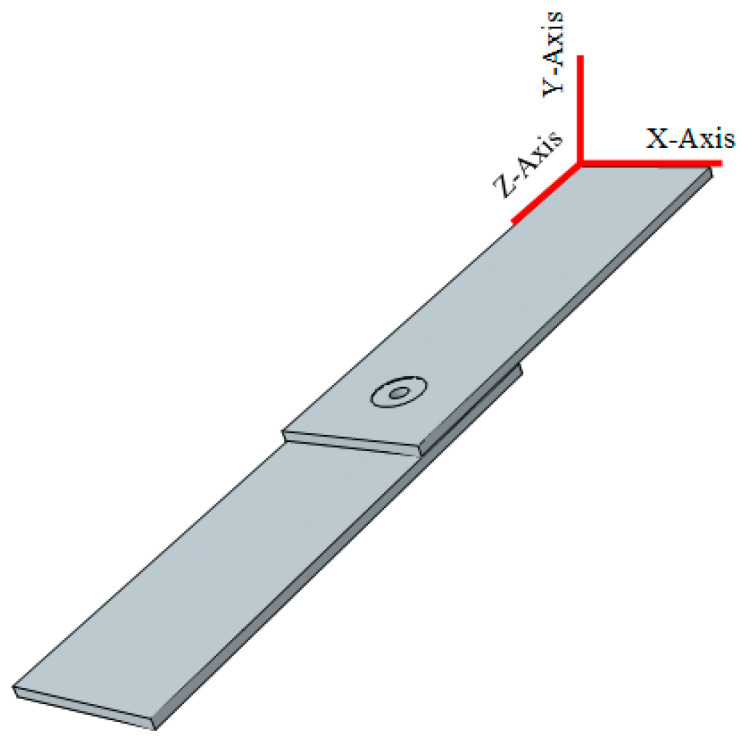
FEM of FSSW joints for structures with a probe hole.

The aluminum plates, joined by friction stir spot welding (FSSW) subjected to static and cyclic load, were analyzed using Abaqus software (V. 6.14, Dassault Systems, Paris, France). The finite element model included three parts: two aluminum sheets and the weld zone (Figure 3).

**Figure 4:**
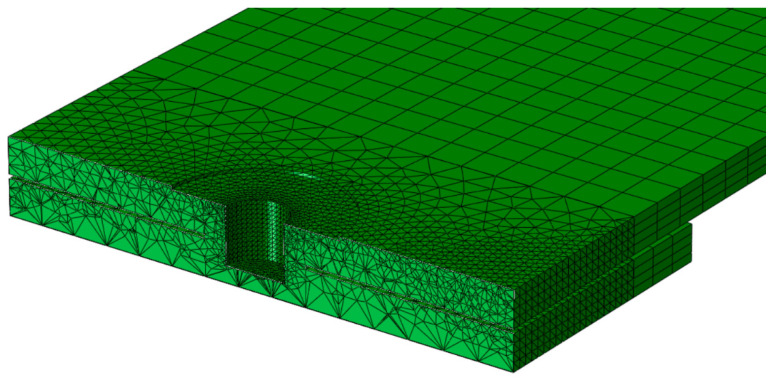
An example of a meshed plate with a probe hole.

The contact between the sheets was assumed to be a perfect bond. Tie constraint types were used to define the perfect bond between the parts (near the weld zone). Figure 4 shows the cross-sectional view of the finite element model of the aluminum plates joined by friction stir spot welding. Three-dimensional 8-node linear hexahedral elements of type C3D8R were used to discretize the regions far away from the welding area. In contrast, quadratic tetrahedron elements of type C3D10 were used to discretize the region around the welding zone [32]. As shown in Figure 4, the mesh near the welding zone was finer to obtain more accurate results. However, in the regions far away from the welding area, a coarser mesh was adopted to reduce the analysis time while keeping the precision intact. Mesh sensitivity analysis was performed to ensure the accuracy of the model. Therefore, the mesh refinement technique was done.

The boundary conditions used for static and fatigue simulations are listed in Table 2. As indicated in Table 2, in order to simulate the static tensile and fatigue tests, the structure is pulled by constant and cyclic displacements, respectively.

### 2.3. Fatigue Life Prediction

The strain-based approach is being widely used to predict fatigue life of different materials. In the present study, stress-life fatigue curves for high-cycle fatigue, as well as stress-strain curves hysteresis loop, are plotted. Total strain amplitude is divided into elastic and plastic strain components based on data from the steady-state hysteresis loops [33]. In this regard, to calculate the steady–state hysteresis loops, the elastic–plastic model is used to model the structure joined by FSSW. By using the hysteresis loop, the plastic strain can be determined by taking the intercept of the loop on the strain axis. The stress-life curve could be linearized by taking life cycles until the failure ($N_{f}$) on a logarithmic scale. The curve could be represented by:

(1) $$\sigma_{a}={\overset{´}{\sigma }}_{f}{\left(2N_{f}\right)}^{b}$$

where ${\overset{´}{\sigma }}_{f}$ is the fatigue strength coefficient and b is the fatigue strength exponent. Coffin and Manson [33] found that the plastic strain-life data could also be linearized by taking $N_{f}$ on a logarithmic scale, and it can be express as:

(2) $$\frac{\Delta \varepsilon_{ave-p}}{2}={\overset{´}{\varepsilon }}_{f}{\left(2N_{f}\right)}^{c}$$

where $\frac{\Delta \varepsilon_{ave-p}}{2}$ is the volume average plastic strain amplitude, ${\overset{´}{\varepsilon }}_{f}$ is the fatigue ductility coefficient, and c is the fatigue ductility exponent.

In the present study, to calculate the strain, homogenized variables (strain) are determined. In this regard, the strain is calculated by taking volume average of the strain from all elements in the cylindrical-shaped volume around the probe hole. This volume is determined to gain more accurate results as the area near the probe hole has a significant effect on both tensile and fatigue failure of the structure.

This specified volume has a diameter equal to the outer clamping ring diameter (10 mm); the height equals the overlap thickness (4 mm), where the hole (or refilled probe hole) is located at the center of the cylindrical-shaped volume. The total strain is calculated as:

(3) $$\varepsilon_{ave}=\frac{1}{V_{m}}\int^{\;}\varepsilon_{m}dV$$

In Equation (3), $\varepsilon_{ave}$ is the volume averaged total strain (consist of elastic and plastic strain), *ε_m_* is the local strain in each element, and *V_m_* is the total volume of the specified cylindrical-shape section. After calculating the volume averaged total strain by Equation (3), the steady-state hysteresis loop is plotted for different applied loads, and then the plastic strain amplitude is determined. By employing Equation (2), the number of cycles to failure is determined for different applied loads and joints with a probe hole as well as with refilled probe holes.

After calculating fatigue life, the results are compared with the experimental data available in the literature [12]. In this research, the material constants are selected from the literature and are listed in Table 3. In order to calculate fatigue life, the hysteresis loop is calculated for the FSSW joint using Abaqus. Then, by developing a Matlab (MathWorks, United States) code, the fatigue life is calculated.

## 3. Results

### 3.1. Tensile Strength

**Figure 5:**
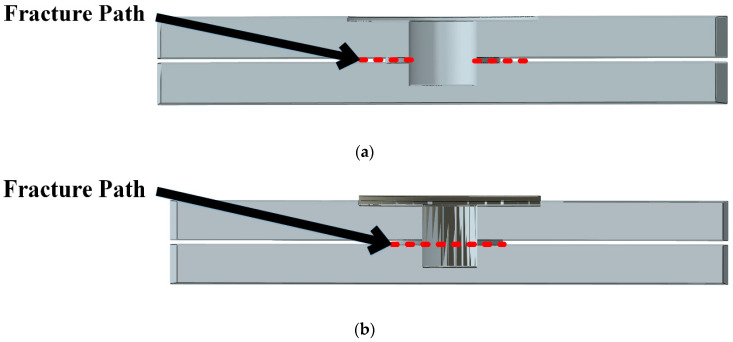
The fracture path for FSSW joint (**a**) with probe hole and (**b**) with refilled probe hole.

**Figure 6:**
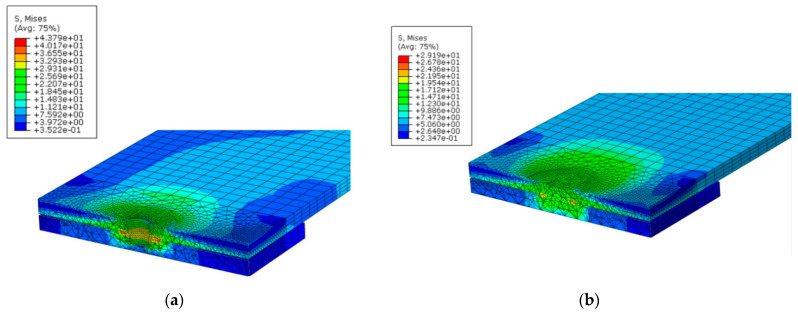
The stress distribution around the hole in a model with (**a**) probe hole and (**b**) refilled probe hole (stresses are in MPa).

Figure 5 illustrates the fracture surfaces between the upper and lower aluminum plates at the joints with the probe hole (Figure 5a) and the refilled probe hole (Figure 5b). As shown in this figure, the upper and lower plates are tied to each other only near the weld zone. By increasing the load in the tensile test, the stress increases, and failure occurs. In the present study, the maximum stress criterion is employed to investigate the failure under static loading. In the present study, the effect of a geometrical defect in the upper and lower sheet interface due to the penetrating of the tool in the lower sheet, which is called hook formation [35], is not considered. The stress distribution around the hole area in the FEM model for FSSW with probe hole under tensile loading is demonstrated in Figure 6a. As shown in this figure, the maximum stress level occurs in the fracture path around the hole. Figure 6b shows the stress distribution around the refilled welded area, which shows that after re-filling the hole, the maximum stress decreases (by 33%) in the fracture surface direction (compared to Figure 6a) at the same applied load. As shown in Figure 5a,b, the effective fracture path (red dotted dash line) width in the structure with a refilled probe hole is larger than the effective fracture path in the structure with a probe hole, which results in higher tensile strength.

The tensile strength of the joints with probe hole and re-filled probe holes obtained from the current FEM model are reported in Table 4 and are compared to the experimental results of Uematsu et al. [12]. From Table 4, it could be perceived that the FEM results are in good agreement with the experimental data, and both studies show that re-filling the probe hole increases the tensile strength. It is worth mentioning that the possible reasons for the numerical difference between the present study and experimental results [12] can be attributed to unknown properties of the welded zone, especially the heat-affected zone (HAZ), as well as ignoring the hook formation effect on tensile strength.

### 3.2. Fatigue Prediction

**Figure 7:**
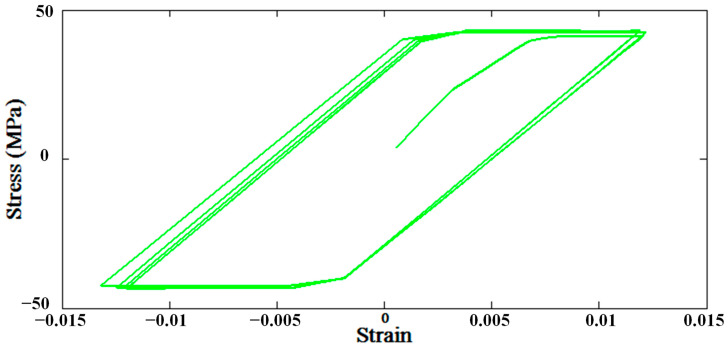
The hysteresis loop for 10 cycles and R = −1.

In order to predict the fatigue life of FSSW joints, first, the hysteresis loop is determined to calculate the plastic strain amplitude. Afterward, the fatigue life is predicted employing the Coffin-Manson Equation, and then it is compared to experimental data [12]. Figure 7 shows the hysteresis loop obtained by Abaqus software for the FSSW joint with a re-filled probe hole for a stress ratio of R = −1, which is defined as the ratio of minimum stress to maximum stress.

**Figure 8:**
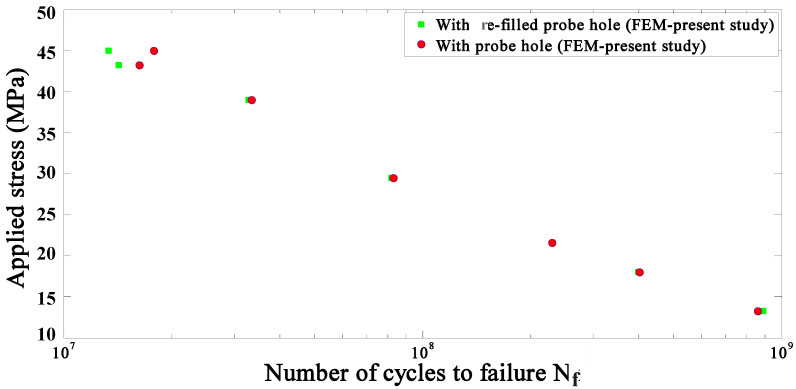
The fatigue life for different maximum stress applied to structures far away from the welding area for R = 0.1.

Available experimental data [12] is reported for R = 0.1, so in the present study, fatigue is predicted for R = 0.1. Figure 8 shows the relationship between the maximum applied stress, $\sigma_{f}$, and the number of cycles to failure ($N_{f}$) in the joints with a probe hole and with a re-filled probe hole for R = 0.1, as predicted by FEM. While the fatigue strengths of both joint types are almost identical at low applied stress levels, the joint without a probe hole has slightly higher fatigue strength than the joint with a probe hole. At high applied stress levels (45 MPa), the joint with the probe hole has a higher fatigue strength. Therefore, at high-stress levels, not only the re-filling process does not increase the fatigue life, but it actually decreases it. Available experimental data [12] have the same trend as the FEM results presented in this study. In the present study, stress and strain (hysteresis loop) are identified near the critical zone in the high-stress concentration area close to the fracture path, which is the same as the experimental observation [12]. This approach helps to obtain reliable FEM results. Therefore, there is a good agreement between FEM results and available experimental data.

**Figure 9:**
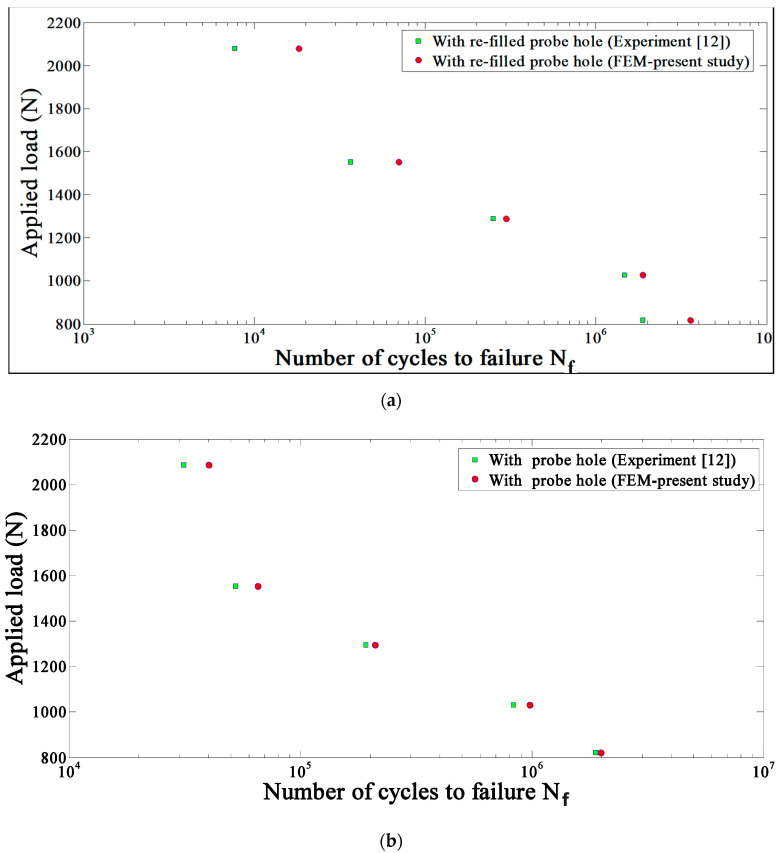
The maximum load applied to the structures versus the number of cycles to failure for both the experimental [12] and FEM studies with (**a**) re-filled probe hole and (**b**) with probe hole for R = 0.1.

Figure 9 compares the numerical (present study) and experimental [12] results for the FSSW joints with the refilled (Figure 9a) and unfilled (Figure 9b) probe holes. These figures indicate the relationship between the maximum load ($P_{max}$) applied to structure and the number of cycles to failure, $N_{f}$, for both the experimental and numerical analysis. As shown in these figures, there is a good agreement between experimental and FEM results. However, the discrepancy between numerical results and available experimental data [12] may be due to the fact that the hook formation was not taken into account. In the FEM analysis, the refilled parts properties are assumed to be the same as the properties of the parent plate (aluminum 6061-T4), and hence the refilled section is part of the parent plate; therefore, the FEM predicts higher fatigue life. By comparing Figure 9a, 9b at a lower applied load, it can be seen that re-filling the probe hole enhances the fatigue life. It is also shown that the result of both the FEM and experimental studies [12] have the same trend, where the fatigue strength of both joints are almost the same at low applied loads. Alternatively, at high applied loads, the joint with a probe hole has a slightly higher fatigue strength.

## 4. Conclusions

In the present study, the static tensile strength and fatigue life were computed by performing 3D FEM based on a strain-based approach. The results were validated against the available experimental data in the literature. The results showed that:

- The tensile strength of the FSSW joint is improved by re-filling the probe hole because of the effective cross-sectional area. In fact, the fracture path is increased, and therefore stress in the fracture path decreases.
- Fatigue strengths of joints with both probe hole and re-filling probe hole are almost the same at low applied load levels.
- At high applied load levels, the joint with a probe hole has higher fatigue strength than the joint with a re-filled probe hole.
- Using a strain-based approach, by calculating the strain from volume averaging of the strains of the elements near the welding zone (a cylindrical-shaped volume), is a reliable approach to determine the fatigue life.

In this paper, a model based on FEM analysis and strain-based model was proposed to study the FSSW joints and the effect of re-filling probe holes on the fatigue life improvement of the FSSW joints. The results were in good agreement with available experimental data. Therefore, the proposed model is a reliable model and it could be used in future studies or even in the industry to examine the effect of other parameters (such as hole diameter, thermal history, etc.) on tensile strength and fatigue life of FSSW joints.

## Figures and Tables

**Table 1:** Johnson-Cook constants of A6061 [29].

A (MPa)	B (MPa)	C_0_	n	m_0_
250	79.7	0.00249	0.499	1.499

**Table 2:** Boundary conditions.

	Plane	Z = 0	Y = 0	X = 0	Z = 2L
Simulation					
Static		$U_{z}=0$	$U_{y}=0$	$U_{x}=0$	$U_{z}\;\neq \;0$, $F_{z}=constant$
Fatigue		$U_{z}=0$	$U_{y}=0$	$U_{x}=0$	$U_{z}\;\neq \;0$, $F_{z}=cyclic$

**Table 3:** Fatigue materials constants for aluminum 6061-T4 [34].

Materials Constant	Aluminum 6061-T4
$\mathrm{Fatigue}\;\mathrm{ductility}\;\mathrm{coefficient},\;{\overset{´}{\varepsilon }}_{f}$ (mm/mm)	0.15
Fatigue ductility exponent, c	−0.520
Fatigue strength coefficient,$\;{\overset{´}{\sigma }}_{f}$ (MPa)	332
Fatigue strength exponent, b	−0.120

**Table 4:** The tensile strength for FSSW joints.

Mechanical Properties	With a Probe Hole		Re-Filled Probe Hole	
	FEM (Present Study)	Experiment [12]	FEM (Present Study)	Experiment [12]
Tensile strength (N)	2950	2654	3621	3458

## Data Availability

Data is contained within the article.
